# Crystal structure of benz­yl(meth­yl)phen­yl[(piperidin-1-ium-1-yl)meth­yl]silane bromide

**DOI:** 10.1107/S2056989015016965

**Published:** 2015-09-17

**Authors:** Eva Rebecca Barth, Christopher Golz, Stephan G. Koller, Carsten Strohmann

**Affiliations:** a Fakultät für Chemie und Chemische Biologie, Technische Universität Dortmund, Otto-Hahn-Strasse 6, 44227 Dortmund, Germany

**Keywords:** crystal structure, chiral organosilane, N—H⋯Br hydrogen bond

## Abstract

The title compound, C_20_H_29_NSi^+^·Br^−^, contains a chiral silicon atom but crystallizes as a racemate. The C—Si—C bond angles in the range of 103.64 (8)–111.59 (9)° are usual for tetra­hedral geometry. The piperidine ring shows a regular chair conformation with an equatorially positioned exocyclic N—C bond. In the crystal, there is a hydrogen bond between the ammonium cation and the bromide anion. The crystal packing shows the dominant inter­molecular inter­action to be the electrostatic attraction between the ammonium cation and the bromide anion.

## Related literature   

Benzyl­meth­yl(piperidino­meth­yl)silane and its methyl­iodide salt are used as model systems to investigate the stereochemistry of substitution reactions with silyllithium compounds as nucleophiles, see: Strohmann *et al.* (2004 ▸).
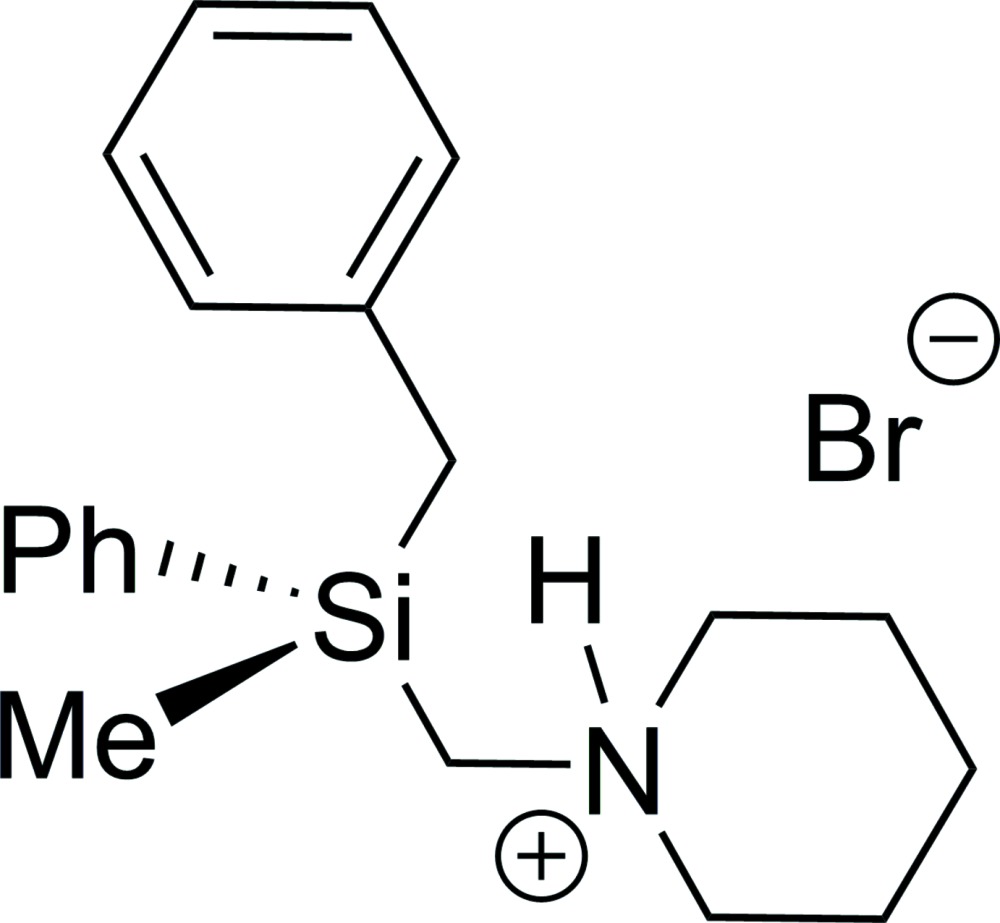


## Experimental   

### Crystal data  

C_20_H_28_NSi^+^·Br^−^
*M* *_r_* = 390.43
Monoclinic, 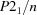

*a* = 13.9311 (12) Å
*b* = 7.4605 (6) Å
*c* = 19.3515 (17) Å
β = 100.926 (2)°
*V* = 1974.8 (3) Å^3^
*Z* = 4
Mo *K*α radiation
μ = 2.14 mm^−1^
*T* = 173 K
0.2 × 0.2 × 0.1 mm

### Data collection  

Bruker APEXII CCD diffractometer
Absorption correction: multi-scan (*SADABS*, Bruker, 2015 ▸) *T*_min_ = 0.421, *T*_max_ = 0.746
26749 measured reflections
4759 independent reflections
3948 reflections with *I* > 2σ(*I*)
*R*_int_ = 0.040

### Refinement  

*R*[*F* ^2^ > 2σ(*F* ^2^)] = 0.031
*wR*(*F* ^2^) = 0.080
*S* = 1.02
4759 reflections
213 parameters
H atoms treated by a mixture of independent and constrained refinement
Δρ_max_ = 0.66 e Å^−3^
Δρ_min_ = −0.25 e Å^−3^

### 

Data collection: *APEX2* (Bruker, 2003 ▸); cell refinement: *SAINT* (Bruker, 2003 ▸); data reduction: *SAINT*; program(s) used to solve structure: *SHELXT* (Sheldrick, 2015*a* ▸); program(s) used to refine structure: *SHELXL2014* (Sheldrick, 2015*b* ▸); molecular graphics: *SHELXTL* (Sheldrick, 2008 ▸); software used to prepare material for publication: *OLEX2* (Dolomanov *et al.*, 2009 ▸) and *publCIF* (Westrip, 2010 ▸).

## Supplementary Material

Crystal structure: contains datablock(s) I. DOI: 10.1107/S2056989015016965/sj5471sup1.cif

Structure factors: contains datablock(s) I. DOI: 10.1107/S2056989015016965/sj5471Isup2.hkl

Supporting information file. DOI: 10.1107/S2056989015016965/sj5471Isup3.cml

**Fig. 1.**
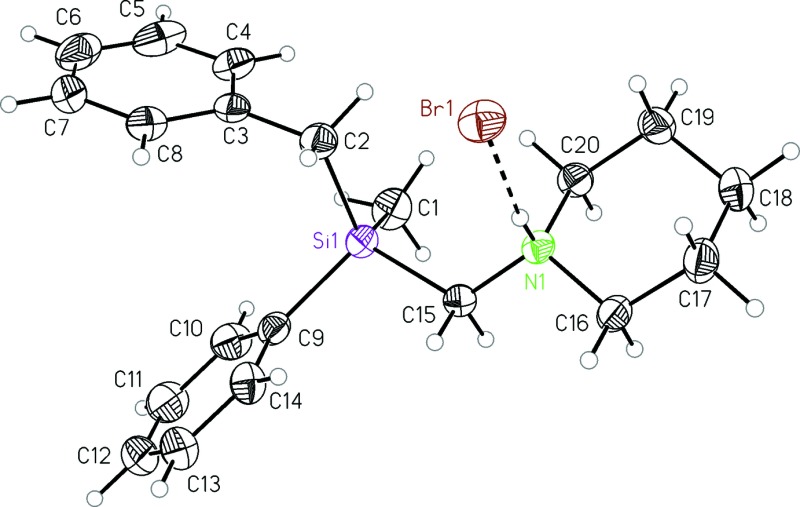
Molecular structure of the title compound with anisotropic displacement ellipsoids drawn at the 50% probability level. An intermolecular hydrogen bond is shown as a dashed line.

**Fig. 2.**
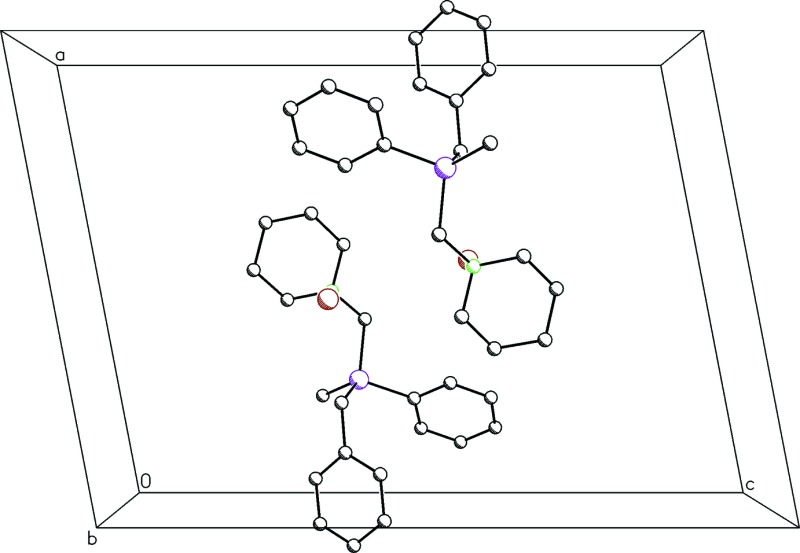
Crystal packing of the title compound viewed along b axis. H-atoms are omitted for clarity.

. DOI: 10.1107/S2056989015016965/sj5471fig1.tif
Mol­ecular structure of the title compound with anisotropic displacement ellipsoids drawn at the 50% probability level. An inter­molecular hydrogen bond is shown as a dashed line.

b . DOI: 10.1107/S2056989015016965/sj5471fig2.tif
Crystal packing of the title compound viewed along *b* axis. H-atoms are omitted for clarity.

CCDC reference: 1423495

Additional supporting information:  crystallographic information; 3D view; checkCIF report

## Figures and Tables

**Table 1:** Hydrogen-bond geometry (, )

*D*H*A*	*D*H	H*A*	*D* *A*	*D*H*A*
N1H1Br1	0.82(2)	2.41(2)	3.2242(15)	173.4(17)

## supplementary crystallographic information

### S1. Synthesis and crystallization

The title compound was synthesized to increase the enanti­omeric ratio of the chiral benzyl­methyl­phenyl­(piperidino­methyl)­silane. Therefore the silane was treated with 1 eq of HBr in Et_2_O (1 M). Solvent was evaporated in vacuum, and the solid residue was recrystallized from iso­propanol at 243 K for 24 h. The crystals were washed with cold iso­propanol to prepare them for X-ray analysis.

### S2. Refinement

Crystal data, data collection and structure refinement details are summarized in Table 1. Treatment of hydrogen atoms: *U*_iso_(H) = 1.2 times *U*_iso_(C) for CH and CH_2_, *U*_iso_(H) = 1.5 times *U*_iso_(C) for CH_3_; refinement: secondary CH_2_ and aromatic H with riding coordinates, CH_3_ as a rotating methyl group.

### Crystal data

**Table:**

C_20_H_28_NSi^+^·Br^−^	*F*(000) = 816
*M**_r_* = 390.43	*D*_x_ = 1.313 Mg m^−^^3^
Monoclinic, *P*2_1_/*n*	Mo *K*α radiation, λ = 0.71073 Å
*a* = 13.9311 (12) Å	Cell parameters from 7370 reflections
*b* = 7.4605 (6) Å	θ = 2.9–27.9°
*c* = 19.3515 (17) Å	µ = 2.14 mm^−^^1^
β = 100.926 (2)°	*T* = 173 K
*V* = 1974.8 (3) Å^3^	Block, colourless
*Z* = 4	0.2 × 0.2 × 0.1 mm

### Data collection

**Table:**

Bruker APEXII CCD diffractometer	3948 reflections with *I* > 2σ(*I*)
φ and ω scans	*R*_int_ = 0.040
Absorption correction: multi-scan (*SADABS*, Bruker, 2015)	θ_max_ = 28.0°, θ_min_ = 2.1°
*T*_min_ = 0.421, *T*_max_ = 0.746	*h* = −18→18
26749 measured reflections	*k* = −9→9
4759 independent reflections	*l* = −25→25

### Refinement

**Table:**

Refinement on *F*^2^	Primary atom site location: structure-invariant direct methods
Least-squares matrix: full	Hydrogen site location: mixed
*R*[*F*^2^ > 2σ(*F*^2^)] = 0.031	H atoms treated by a mixture of independent and constrained refinement
*wR*(*F*^2^) = 0.080	*w* = 1/[σ^2^(*F*_o_^2^) + (0.0462*P*)^2^ + 0.3152*P*] where *P* = (*F*_o_^2^ + 2*F*_c_^2^)/3
*S* = 1.02	(Δ/σ)_max_ = 0.001
4759 reflections	Δρ_max_ = 0.66 e Å^−^^3^
213 parameters	Δρ_min_ = −0.25 e Å^−^^3^
0 restraints	

### Special details

**Table:**

Experimental. Absorption correction: SADABS-2014/5 (Bruker,2014/5) was used for absorption correction. wR2(int) was 0.1354 before and 0.0450 after correction. The Ratio of minimum to maximum transmission is 0.5649. The λ/2 correction factor is 0.00150.
Geometry. All e.s.d.'s (except the e.s.d. in the dihedral angle between two l.s. planes) are estimated using the full covariance matrix. The cell e.s.d.'s are taken into account individually in the estimation of e.s.d.'s in distances, angles and torsion angles; correlations between e.s.d.'s in cell parameters are only used when they are defined by crystal symmetry. An approximate (isotropic) treatment of cell e.s.d.'s is used for estimating e.s.d.'s involving l.s. planes.
Refinement. 1. Fixed *U*_iso_ At 1.2 times of: All C(H) groups, All C(H,H) groups At 1.5 times of: All C(H,H,H) groups 2.a Secondary CH2 refined with riding coordinates: C2(H2A,H2B), C15(H15A,H15B), C16(H16A,H16B), C17(H17A,H17B), C18(H18A,H18B), C19(H19A,H19B), C20(H20A,H20B) 2.b Aromatic/amide H refined with riding coordinates: C4(H4), C5(H5), C6(H6), C7(H7), C8(H8), C10(H10), C11(H11), C12(H12), C13(H13), C14(H14) 2.c Idealized Me refined as rotating group: C1(H1A,H1B,H1C)

### Fractional atomic coordinates and isotropic or equivalent isotropic displacement parameters (Å^2^)

**Table:**

	*x*	*y*	*z*	*U*_iso_*/*U*_eq_	
Si1	0.27017 (3)	0.16579 (6)	0.40241 (2)	0.02506 (11)	
N1	0.47332 (10)	0.2487 (2)	0.38731 (7)	0.0226 (3)	
H1	0.4648 (13)	0.358 (3)	0.3882 (9)	0.026 (5)*	
C1	0.22693 (15)	−0.0080 (3)	0.33465 (11)	0.0397 (5)	
H1A	0.2388	0.0327	0.2889	0.060*	
H1B	0.1568	−0.0282	0.3317	0.060*	
H1C	0.2624	−0.1202	0.3477	0.060*	
C2	0.22659 (14)	0.3967 (3)	0.37105 (10)	0.0332 (4)	
H2A	0.2580	0.4872	0.4055	0.040*	
H2B	0.2463	0.4217	0.3255	0.040*	
C3	0.11714 (13)	0.4124 (2)	0.36237 (9)	0.0287 (4)	
C4	0.05486 (15)	0.3325 (2)	0.30609 (11)	0.0365 (4)	
H4	0.0816	0.2690	0.2716	0.044*	
C5	−0.04527 (17)	0.3443 (3)	0.29963 (13)	0.0494 (6)	
H5	−0.0867	0.2876	0.2612	0.059*	
C6	−0.08555 (16)	0.4373 (3)	0.34831 (14)	0.0533 (6)	
H6	−0.1546	0.4453	0.3435	0.064*	
C7	−0.02525 (17)	0.5190 (3)	0.40435 (12)	0.0499 (6)	
H7	−0.0528	0.5845	0.4379	0.060*	
C8	0.07535 (15)	0.5058 (3)	0.41163 (10)	0.0376 (4)	
H8	0.1163	0.5609	0.4507	0.045*	
C9	0.22505 (12)	0.1129 (2)	0.48553 (9)	0.0274 (4)	
C10	0.15034 (14)	−0.0107 (3)	0.48712 (11)	0.0375 (4)	
H10	0.1224	−0.0726	0.4452	0.045*	
C11	0.11604 (16)	−0.0451 (3)	0.54855 (12)	0.0463 (5)	
H11	0.0652	−0.1301	0.5482	0.056*	
C12	0.15485 (16)	0.0426 (3)	0.60973 (11)	0.0466 (5)	
H12	0.1311	0.0186	0.6517	0.056*	
C13	0.22922 (17)	0.1669 (3)	0.60999 (11)	0.0423 (5)	
H13	0.2564	0.2284	0.6521	0.051*	
C14	0.26369 (15)	0.2009 (3)	0.54857 (10)	0.0339 (4)	
H14	0.3147	0.2859	0.5493	0.041*	
C15	0.40836 (12)	0.1599 (2)	0.43050 (9)	0.0250 (3)	
H15A	0.4242	0.2126	0.4783	0.030*	
H15B	0.4278	0.0322	0.4352	0.030*	
C16	0.57838 (13)	0.2155 (3)	0.41985 (9)	0.0296 (4)	
H16A	0.5913	0.0849	0.4209	0.036*	
H16B	0.5911	0.2596	0.4690	0.036*	
C17	0.64701 (14)	0.3085 (3)	0.37913 (10)	0.0351 (4)	
H17A	0.7155	0.2796	0.4007	0.042*	
H17B	0.6385	0.4399	0.3818	0.042*	
C18	0.62716 (14)	0.2504 (3)	0.30259 (10)	0.0369 (4)	
H18A	0.6428	0.1216	0.2994	0.044*	
H18B	0.6695	0.3190	0.2764	0.044*	
C19	0.52035 (14)	0.2826 (3)	0.26998 (9)	0.0319 (4)	
H19A	0.5070	0.4130	0.2682	0.038*	
H19B	0.5071	0.2364	0.2211	0.038*	
C20	0.45269 (13)	0.1907 (2)	0.31190 (9)	0.0276 (4)	
H20A	0.3840	0.2195	0.2908	0.033*	
H20B	0.4611	0.0592	0.3094	0.033*	
Br1	0.45737 (2)	0.67990 (2)	0.38635 (2)	0.03319 (7)	

### Atomic displacement parameters (Å^2^)

**Table:**

	*U*^11^	*U*^22^	*U*^33^	*U*^12^	*U*^13^	*U*^23^
Si1	0.0243 (2)	0.0241 (2)	0.0264 (2)	0.00079 (18)	0.00388 (18)	−0.00076 (18)
N1	0.0252 (8)	0.0172 (6)	0.0254 (7)	0.0034 (5)	0.0050 (6)	−0.0016 (5)
C1	0.0374 (11)	0.0395 (11)	0.0399 (10)	−0.0018 (9)	0.0013 (8)	−0.0105 (9)
C2	0.0316 (10)	0.0306 (9)	0.0383 (10)	0.0021 (8)	0.0090 (8)	0.0082 (8)
C3	0.0312 (10)	0.0260 (8)	0.0294 (9)	0.0031 (7)	0.0072 (7)	0.0102 (7)
C4	0.0406 (11)	0.0298 (10)	0.0364 (10)	0.0061 (8)	−0.0001 (8)	0.0057 (8)
C5	0.0436 (13)	0.0386 (12)	0.0582 (14)	−0.0035 (9)	−0.0100 (11)	0.0160 (10)
C6	0.0292 (11)	0.0587 (14)	0.0730 (16)	0.0058 (10)	0.0125 (11)	0.0356 (13)
C7	0.0494 (13)	0.0592 (14)	0.0475 (12)	0.0228 (11)	0.0256 (11)	0.0215 (11)
C8	0.0427 (11)	0.0385 (10)	0.0316 (9)	0.0089 (9)	0.0068 (8)	0.0074 (8)
C9	0.0250 (9)	0.0249 (8)	0.0323 (9)	0.0042 (7)	0.0058 (7)	0.0044 (7)
C10	0.0333 (10)	0.0359 (10)	0.0420 (10)	−0.0051 (8)	0.0037 (8)	0.0071 (8)
C11	0.0355 (11)	0.0475 (12)	0.0578 (14)	−0.0038 (9)	0.0137 (10)	0.0193 (10)
C12	0.0447 (12)	0.0572 (13)	0.0427 (12)	0.0113 (10)	0.0200 (10)	0.0164 (10)
C13	0.0456 (12)	0.0479 (13)	0.0355 (10)	0.0088 (9)	0.0130 (9)	−0.0018 (9)
C14	0.0339 (10)	0.0333 (10)	0.0361 (10)	0.0014 (8)	0.0110 (8)	−0.0032 (8)
C15	0.0254 (9)	0.0248 (8)	0.0251 (8)	0.0012 (6)	0.0052 (6)	0.0018 (6)
C16	0.0243 (9)	0.0356 (9)	0.0280 (9)	0.0052 (7)	0.0028 (7)	−0.0006 (7)
C17	0.0246 (9)	0.0437 (11)	0.0375 (10)	0.0002 (8)	0.0075 (8)	−0.0023 (8)
C18	0.0331 (11)	0.0444 (11)	0.0362 (10)	0.0042 (9)	0.0144 (8)	−0.0007 (9)
C19	0.0365 (10)	0.0340 (9)	0.0264 (8)	0.0038 (8)	0.0091 (7)	0.0000 (7)
C20	0.0302 (9)	0.0286 (9)	0.0234 (8)	0.0022 (7)	0.0035 (7)	−0.0030 (7)
Br1	0.04173 (13)	0.02011 (10)	0.03668 (11)	0.00323 (7)	0.00476 (8)	−0.00210 (7)

### Geometric parameters (Å, º)

**Table:**

Si1—C1	1.8614 (19)	C9—C14	1.400 (3)
Si1—C2	1.8883 (19)	C10—H10	0.9500
Si1—C9	1.8766 (18)	C10—C11	1.387 (3)
Si1—C15	1.8991 (18)	C11—H11	0.9500
N1—H1	0.82 (2)	C11—C12	1.371 (3)
N1—C15	1.498 (2)	C12—H12	0.9500
N1—C16	1.500 (2)	C12—C13	1.389 (3)
N1—C20	1.497 (2)	C13—H13	0.9500
C1—H1A	0.9800	C13—C14	1.387 (3)
C1—H1B	0.9800	C14—H14	0.9500
C1—H1C	0.9800	C15—H15A	0.9900
C2—H2A	0.9900	C15—H15B	0.9900
C2—H2B	0.9900	C16—H16A	0.9900
C2—C3	1.506 (3)	C16—H16B	0.9900
C3—C4	1.391 (3)	C16—C17	1.518 (3)
C3—C8	1.394 (3)	C17—H17A	0.9900
C4—H4	0.9500	C17—H17B	0.9900
C4—C5	1.379 (3)	C17—C18	1.518 (3)
C5—H5	0.9500	C18—H18A	0.9900
C5—C6	1.373 (4)	C18—H18B	0.9900
C6—H6	0.9500	C18—C19	1.521 (3)
C6—C7	1.382 (4)	C19—H19A	0.9900
C7—H7	0.9500	C19—H19B	0.9900
C7—C8	1.385 (3)	C19—C20	1.519 (3)
C8—H8	0.9500	C20—H20A	0.9900
C9—C10	1.395 (3)	C20—H20B	0.9900
			
C1—Si1—C2	111.59 (9)	C10—C11—H11	119.7
C1—Si1—C9	109.93 (9)	C12—C11—C10	120.6 (2)
C1—Si1—C15	111.14 (8)	C12—C11—H11	119.7
C2—Si1—C15	111.09 (8)	C11—C12—H12	120.2
C9—Si1—C2	109.14 (8)	C11—C12—C13	119.55 (19)
C9—Si1—C15	103.64 (8)	C13—C12—H12	120.2
C15—N1—H1	108.7 (13)	C12—C13—H13	120.1
C15—N1—C16	109.70 (13)	C14—C13—C12	119.9 (2)
C16—N1—H1	106.7 (13)	C14—C13—H13	120.1
C20—N1—H1	107.8 (12)	C9—C14—H14	119.3
C20—N1—C15	113.03 (13)	C13—C14—C9	121.48 (19)
C20—N1—C16	110.67 (13)	C13—C14—H14	119.3
Si1—C1—H1A	109.5	Si1—C15—H15A	107.0
Si1—C1—H1B	109.5	Si1—C15—H15B	107.0
Si1—C1—H1C	109.5	N1—C15—Si1	121.12 (11)
H1A—C1—H1B	109.5	N1—C15—H15A	107.0
H1A—C1—H1C	109.5	N1—C15—H15B	107.0
H1B—C1—H1C	109.5	H15A—C15—H15B	106.8
Si1—C2—H2A	109.4	N1—C16—H16A	109.3
Si1—C2—H2B	109.4	N1—C16—H16B	109.3
H2A—C2—H2B	108.0	N1—C16—C17	111.52 (14)
C3—C2—Si1	111.32 (12)	H16A—C16—H16B	108.0
C3—C2—H2A	109.4	C17—C16—H16A	109.3
C3—C2—H2B	109.4	C17—C16—H16B	109.3
C4—C3—C2	121.46 (17)	C16—C17—H17A	109.4
C4—C3—C8	118.03 (18)	C16—C17—H17B	109.4
C8—C3—C2	120.50 (17)	C16—C17—C18	111.09 (16)
C3—C4—H4	119.5	H17A—C17—H17B	108.0
C5—C4—C3	120.9 (2)	C18—C17—H17A	109.4
C5—C4—H4	119.5	C18—C17—H17B	109.4
C4—C5—H5	119.7	C17—C18—H18A	109.7
C6—C5—C4	120.5 (2)	C17—C18—H18B	109.7
C6—C5—H5	119.7	C17—C18—C19	110.05 (15)
C5—C6—H6	120.2	H18A—C18—H18B	108.2
C5—C6—C7	119.7 (2)	C19—C18—H18A	109.7
C7—C6—H6	120.2	C19—C18—H18B	109.7
C6—C7—H7	120.0	C18—C19—H19A	109.4
C6—C7—C8	120.1 (2)	C18—C19—H19B	109.4
C8—C7—H7	120.0	H19A—C19—H19B	108.0
C3—C8—H8	119.6	C20—C19—C18	111.38 (15)
C7—C8—C3	120.8 (2)	C20—C19—H19A	109.4
C7—C8—H8	119.6	C20—C19—H19B	109.4
C10—C9—Si1	122.04 (14)	N1—C20—C19	111.48 (14)
C10—C9—C14	117.13 (17)	N1—C20—H20A	109.3
C14—C9—Si1	120.80 (14)	N1—C20—H20B	109.3
C9—C10—H10	119.3	C19—C20—H20A	109.3
C11—C10—C9	121.40 (19)	C19—C20—H20B	109.3
C11—C10—H10	119.3	H20A—C20—H20B	108.0
			
Si1—C2—C3—C4	−74.37 (19)	C9—Si1—C2—C3	−55.09 (15)
Si1—C2—C3—C8	104.58 (17)	C9—Si1—C15—N1	−160.77 (12)
Si1—C9—C10—C11	−178.33 (15)	C9—C10—C11—C12	0.2 (3)
Si1—C9—C14—C13	178.18 (15)	C10—C9—C14—C13	0.0 (3)
N1—C16—C17—C18	−56.7 (2)	C10—C11—C12—C13	0.0 (3)
C1—Si1—C2—C3	66.61 (15)	C11—C12—C13—C14	−0.1 (3)
C1—Si1—C9—C10	−15.24 (18)	C12—C13—C14—C9	0.2 (3)
C1—Si1—C9—C14	166.66 (15)	C14—C9—C10—C11	−0.2 (3)
C1—Si1—C15—N1	81.20 (15)	C15—Si1—C2—C3	−168.75 (12)
C2—Si1—C9—C10	107.45 (16)	C15—Si1—C9—C10	−134.11 (15)
C2—Si1—C9—C14	−70.65 (16)	C15—Si1—C9—C14	47.79 (16)
C2—Si1—C15—N1	−43.69 (15)	C15—N1—C16—C17	−178.06 (14)
C2—C3—C4—C5	178.46 (17)	C15—N1—C20—C19	−179.63 (14)
C2—C3—C8—C7	−179.31 (17)	C16—N1—C15—Si1	−177.28 (11)
C3—C4—C5—C6	0.8 (3)	C16—N1—C20—C19	−56.13 (18)
C4—C3—C8—C7	−0.3 (3)	C16—C17—C18—C19	55.3 (2)
C4—C5—C6—C7	−0.3 (3)	C17—C18—C19—C20	−55.1 (2)
C5—C6—C7—C8	−0.6 (3)	C18—C19—C20—N1	56.0 (2)
C6—C7—C8—C3	0.9 (3)	C20—N1—C15—Si1	−53.24 (17)
C8—C3—C4—C5	−0.5 (3)	C20—N1—C16—C17	56.54 (19)

### Hydrogen-bond geometry (Å, º)

**Table:**

*D*—H···*A*	*D*—H	H···*A*	*D*···*A*	*D*—H···*A*
N1—H1···Br1	0.82 (2)	2.41 (2)	3.2242 (15)	173.4 (17)
