# Supplementary figures and images for: Crystal structure of benz­yl(meth­yl)phen­yl[(piperidin-1-ium-1-yl)meth­yl]silane bromide
Source: Acta Crystallogr E Crystallogr Commun. 2015 Sep 17;71(Pt 10):o759. doi: 10.1107/S2056989015016965 (PMC4647413; doi:10.1107/S2056989015016965)

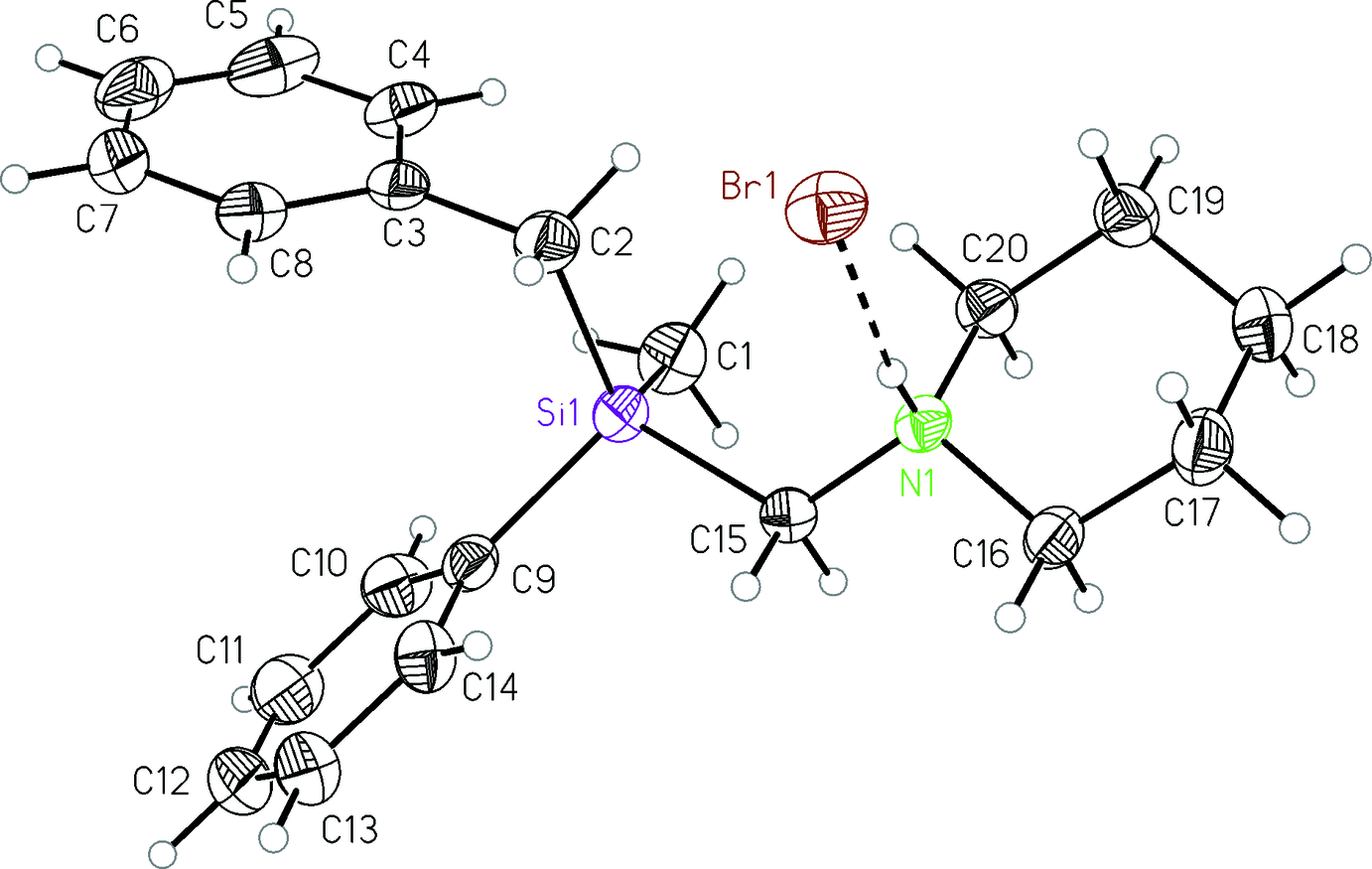

Supplement: Supplementary file 4 [file e-71-0o759-fig1.tif]

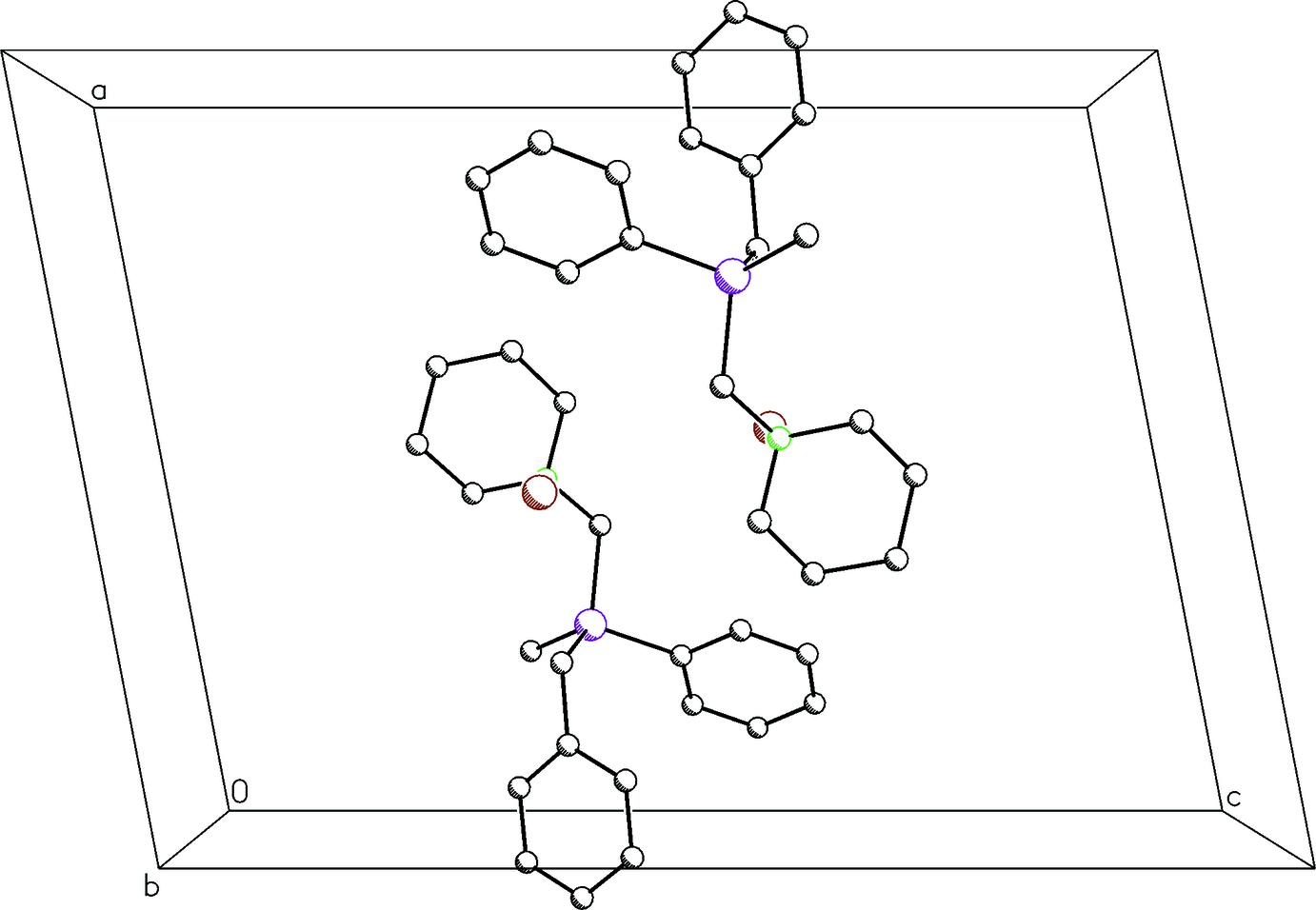

Supplement: Supplementary file 5 [file e-71-0o759-fig2.tif]
